# War related disruption of clinical tuberculosis services in Tigray, Ethiopia during the recent regional conflict: a mixed sequential method study

**DOI:** 10.1186/s13031-024-00583-8

**Published:** 2024-04-10

**Authors:** Kibrom Gebreselasie Gebrehiwot, Gebremedhin Berhe Gebregergis, Measho Gebreslasie Gebregziabher, Teklay Gebrecherkos, Wegen Beyene Tesfamariam, Hailay Gebretnsae, Gebregziabher Berihu, Letebrhan Weldemhret, Goyitom Gebremedhn, Tsegay Wellay, Hadish Bekuretsion, Aregay Gebremedhin, Tesfay Gebregzabher Gebrehiwet, Gebretsadik Berhe

**Affiliations:** 1 School of Medicine, College of Health Science, Mekelle University, PO Box: 1871, Mekelle, Ethiopia; 2 School of Public Health, College of Health Science, Mekelle University, Mekelle, Ethiopia; 3 Tigray Health Research Institute, Mekelle, Tigray, Ethiopia; 4 Tigray Regional Health Bureau, Mekelle, Tigray, Ethiopia

**Keywords:** Tuberculosis, War, Siege, Service disruption, Tigray, Ethiopia

## Abstract

**Background:**

More than 70% of the health facilities in Tigray, northern Ethiopia, have been totally or partially destroyed by the recent war in the region. Diagnosis and management of tuberculosis were among many health services that suffered. In this study we assess the status of tuberculosis care in health facilities of Tigray during the recent war and compare it with the immediate pre-war state.

**Methods:**

Using sequential mixed method, we analyzed and compared the availability of diagnostic services in 69 health facilities and the utilization of tuberculosis care in 50 of them immediately before the war (September-October 2020) and during the war (November-July 2021). TB focal persons in each selected health facility were interviewed to evaluate the status of diagnostic services. Patient service utilization was assessed using health facility registrations. We also compared the average monthly case detection rate of multidrug resistant tuberculosis in the region before and during the war. We computed summary statistics and performed comparisons using t-tests. Finally, existing challenges related to tuberculosis care in the region were explored via in-depth interviews. Two investigators openly coded and analyzed the qualitative data independently via thematic analysis.

**Results:**

Among the 69 health facilities randomly selected, the registers of 19 facilities were destroyed by the war; data from the remaining 50 facilities were included in the TB service utilization analysis. In the first month of the war (November 2021) the number of tuberculosis patients visiting health facilities fell 34%. Subsequently the visitation rate improved steadily, but not to pre-war rates. This reduction was significant in northwest, central and eastern zones. Tuberculosis care in rural areas was hit hardest. Prior to the war 60% of tuberculosis patients were served in rural clinics; this number dropped to an average of 17% during the war. Health facilities were systematically looted. Of the 69 institutions assessed, over 69% of the microscopes in health centers, 87.5% of the microscopes in primary hospitals, and 68% of the microscopes in general hospitals were stolen or damaged. Two GeneXpert nucleic acid amplification machines were also taken from general hospitals. Regarding drug resistant TB, the average number of multidrug resistant tuberculosis (MDR TB) cases detected per month was reduced by 41% during the war with p-value < 0.001. In-depth interviews with eight health care workers indicated that the main factors affecting tuberculosis care in the area were lack of security, health facility destruction, theft of essential equipment, and drug supply disruption.

**Conclusion and recommendation:**

Many tuberculosis patients failed to visit health facilities during the war. There was substantial physical damage to health care facilities and systematic looting of diagnostic equipment. Restoring basic public services and revitalizing clinical care for tuberculosis need urgent consideration.

**Supplementary Information:**

The online version contains supplementary material available at 10.1186/s13031-024-00583-8.

## Introduction

The recent war in Tigray, Ethiopia, devastated the health care system, leaving 85% of the health centers and 70% of the hospitals partially or completely non-functional. They were looted and destroyed by combatants, as reported by Médecins Sans Frontières [1]. Some medical personnel were put to death while others fled or were forcibly relocated. Of 312 ambulances in Tigray, 274 were stolen or destroyed [1, 2]. In addition, the war caused massive civilian displacement, starvation, overcrowding, and the shutdown of basic public services (telecommunication, banking, and transportation). Nearly three million civilians were displaced internally or fled to Sudan [3]. When active fighting decreased, the government imposed a siege. The war created fertile ground for the spread of TB and emergence of drug resistance. Under such conditions, TB-related morbidity usually doubles or triples [4].

In 2020, just before the conflict, there were about 6,697 tuberculosis (TB) patients (including 89 with multidrug-resistant TB), 43,000 people living with HIV/AIDS (PLWHA) patients, and 24,253 diabetes patients on follow-up [5].

We assessed the availability and utilization of TB clinical services in Tigray during the recent war (between November 2020 and July 2021) and compared it with the pre-war state. Furthermore, we assessed the challenges of providing TB clinical services in the region, including the damage to laboratory services. This will hopefully be an input as the region struggles to recover.

## Methods

### Study area

**Fig. 1:**
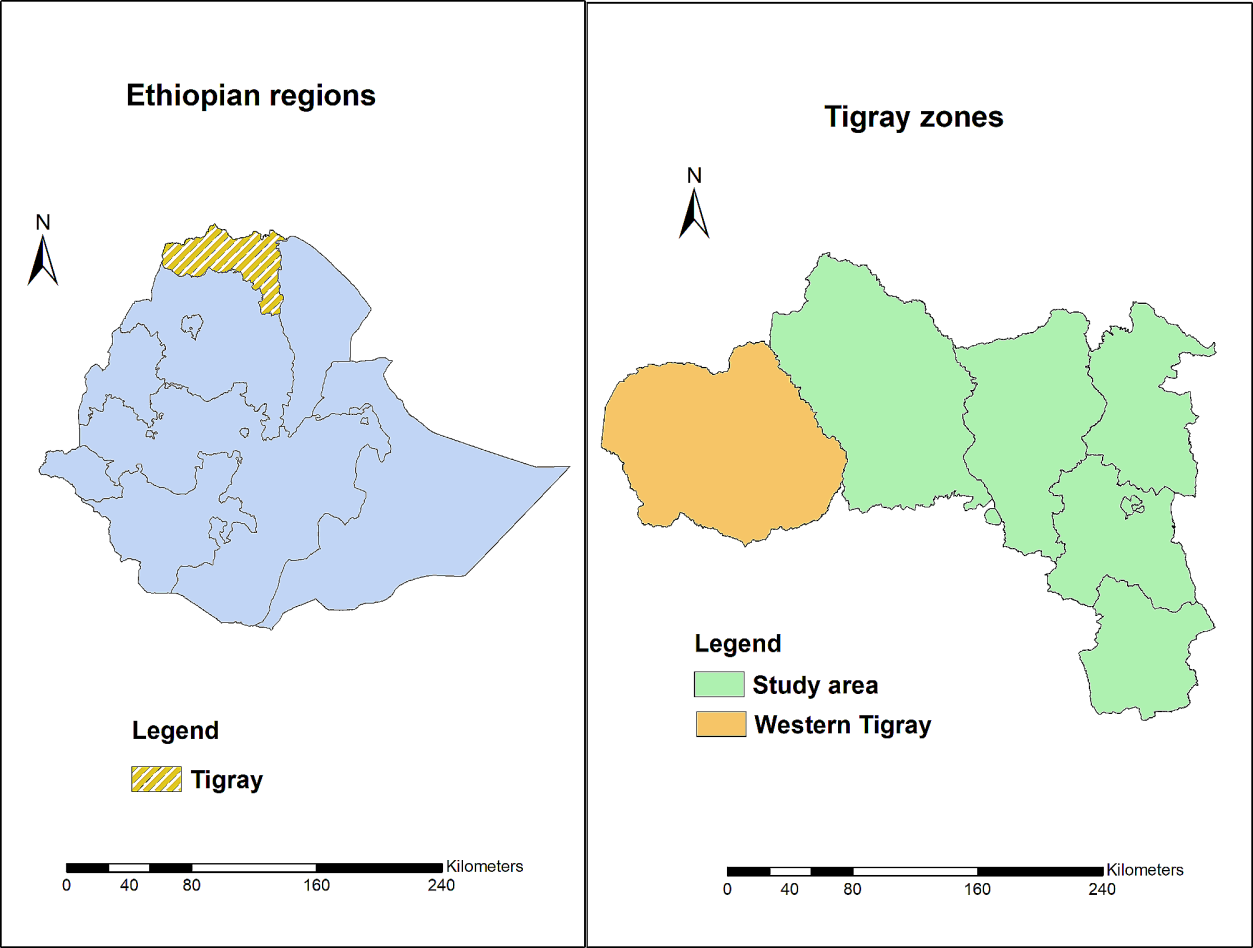
Map of Ethiopia showing the location of Tigray and its different zones

Tigray is one of twelve regional states in Ethiopia. It is situated in the northernmost part of the nation, bordering Eritrea to the north, Sudan to the west, the Amhara region to the south, and the Afar region to the east. As of 2022, the region’s population was estimated at 5.7 million (https://www.citypopulation.de/en/ethiopia/admin/ET01__tigray/). Before the war, Tigray had two referral hospitals, 14 general hospitals, 24 primary hospitals, 231 health centers, and 741 health posts in its 7 zones (Refer to Fig. 1) [8]. TB diagnostic and treatment services were provided free of charge in all public health facilities.

During the study period Western Tigray was in the hands of foreign forces, with reports of ongoing ethnicity-based massacres of civilians. Access to the area was also denied to United Nations investigators and journalists. It was therefore excluded and the study conducted in the remaining six zones.

### Study design and population

We used an explanatory sequential mixed method. First, we collected TB patient data (both susceptible and multidrug resistant forms) from health facility registries and we interviewed TB focal persons to assess laboratory-related damage. Next, we carried out in-depth interviews with TB case team coordinators, TB focal persons, laboratory staff, and medical directors to gather qualitative data regarding the difficulties faced in the diagnosis, management and treatment of TB.

### Sample size determination and sampling procedures

Out of 229 health facilities in six zones, we randomly selected and visited 69 facilities (six general hospitals, twelve primary hospitals, and 51 health centers). Damage assessment related to laboratory diagnostics was possible in all 69 health facilities. However, we were unable to obtain the registers of 19 health institutions, as they were destroyed during the war; thus, TB service utilization data were collected from the 50 health facilities only. Eight in-depth interviews were conducted and saturation of information was used to indicate when to terminate recruitment and interview. The study participants were selected based on their position, professional relevance, and work experience. The participants were from a health center, a referral hospital, a reference laboratory, a district health office, and regional health bureau.

### Data collection procedure and quality assurance

We developed a data abstraction format for the quantitative data collection. This contained the name and location of the health facility (urban or rural), total number of TB patients seen per month (September-July), the number of new MDR TB patients registered in any given month during the study period, and whether the patients received treatment. We used a different data collection tool to determine the amount of diagnostic equipment (microscopes, acid fast stain reagents, microscope slides, and GeneXpert machines) at each medical facility prior to and during the conflict. This data was used to evaluate the damage sustained by individual laboratories.

Health professionals experienced in data collection were recruited for data collection and the study team supervised the process. Four experienced investigators conducted the IDI in private settings with minimum noise. The demographics of respondents, the state of TB-related services in the respondent’s medical facility before and after the war in terms of diagnostics, the sufficiency of supplies, the effectiveness of the health delivery system, the case detection rate, and treatment outcomes were the primary topics covered in the interviews.

One day of training was given to the data collectors and supervisors. A pretest of the instruments was conducted in three health facilities outside of the study setting. Comments forwarded by the discussants and supervisors were incorporated.

### Data analysis

Descriptive statistics were developed using percentages and means with standard deviations. We used a t-test to compare the patient visit data before and during the war. We performed stratified analysis by setting (urban vs. rural) and by zone.

With regard to the qualitative data, each audio-taped interview was reviewed multiple times, transcribed verbatim, and imported into Atlas.ti version 7.5 for coding and analysis. The transcribed and translated versions were read and reread. Filed notes and investigators’ memos were linked to the software. Two investigators openly coded and analyzed the data independently. The investigators held debriefing sessions throughout the data collection and analysis period. Thematic analysis was used; codes were developed based on the original terms used by participants, and then descriptions were subsequently developed followed by identification of the most frequently observed categories and development of themes.

### Ethical clearance

Ethical clearance was obtained from Mekelle University College of Health Sciences Institutional Review Board (Ref.No. MU-IRB 2025/2022). Support letters were obtained from Tigray Health Bureau. In addition, permission was obtained from the head of each health facility before data collection. Verbal informed consent was obtained from each interview participant. Transcripts and voice recordings were kept on a password-protected computer and accessed only by the study team.

## Results

### Quantitative section

Of the 69 institutions included in the study, we were able to analyze patient TB service consumption from 50 of them; as noted above, the registers of 19 health facilities were destroyed during the conflict, including nine from the Northwest zone, six from Central, two from Southeastern, and two from Southern zones 1.

**Table 1:** Number of health facilities included in service consumption analysis

Zone	Health center	Primary hospital	General hospital	Total
Central	1	3	2	6
Eastern	12	2	1	15
Mekelle	2	1	1	4
Northwest	10	1	0	11
Southeast	4	1	0	5
Southern	6	1	2	9
Total	35	9	6	50

**Fig. 2:**
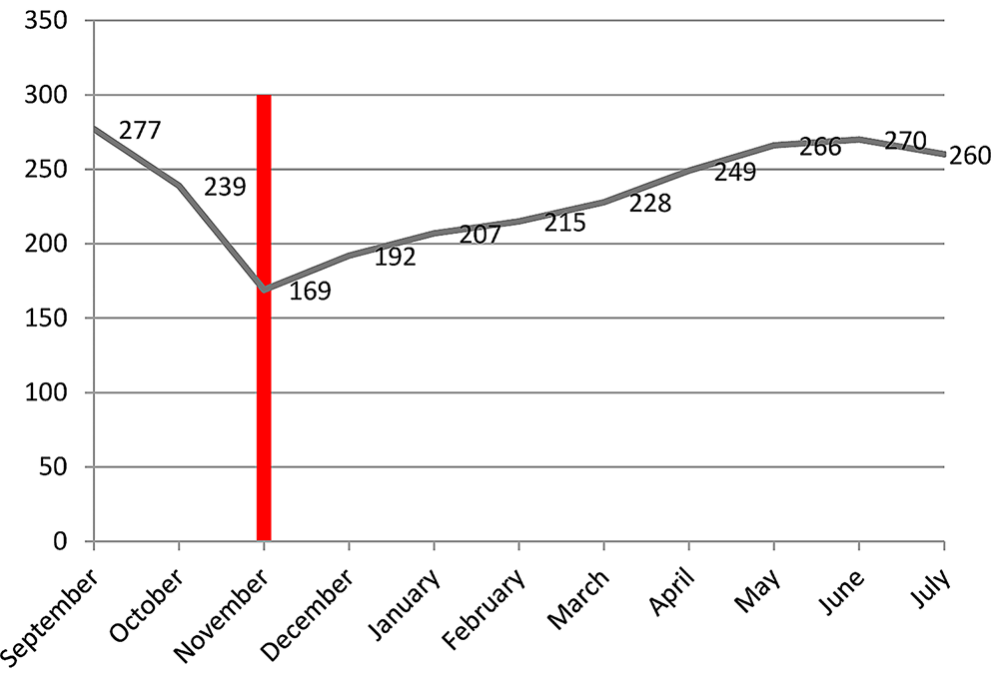
Number of TB patients visiting study health facilities in Tigray before and during the war. The vertical line indicates the start of the war (November 4, 2020)

Pre-war (September-October), an average of 259 TB patients were seen each month in 50 health facilities. After the outbreak of war (November) this dropped to 169, a 34.5% reduction. Over time patients slowly resumed visiting, but the numbers remained lower than pre-war visit rate (Fig. 2).

**Fig. 3:**
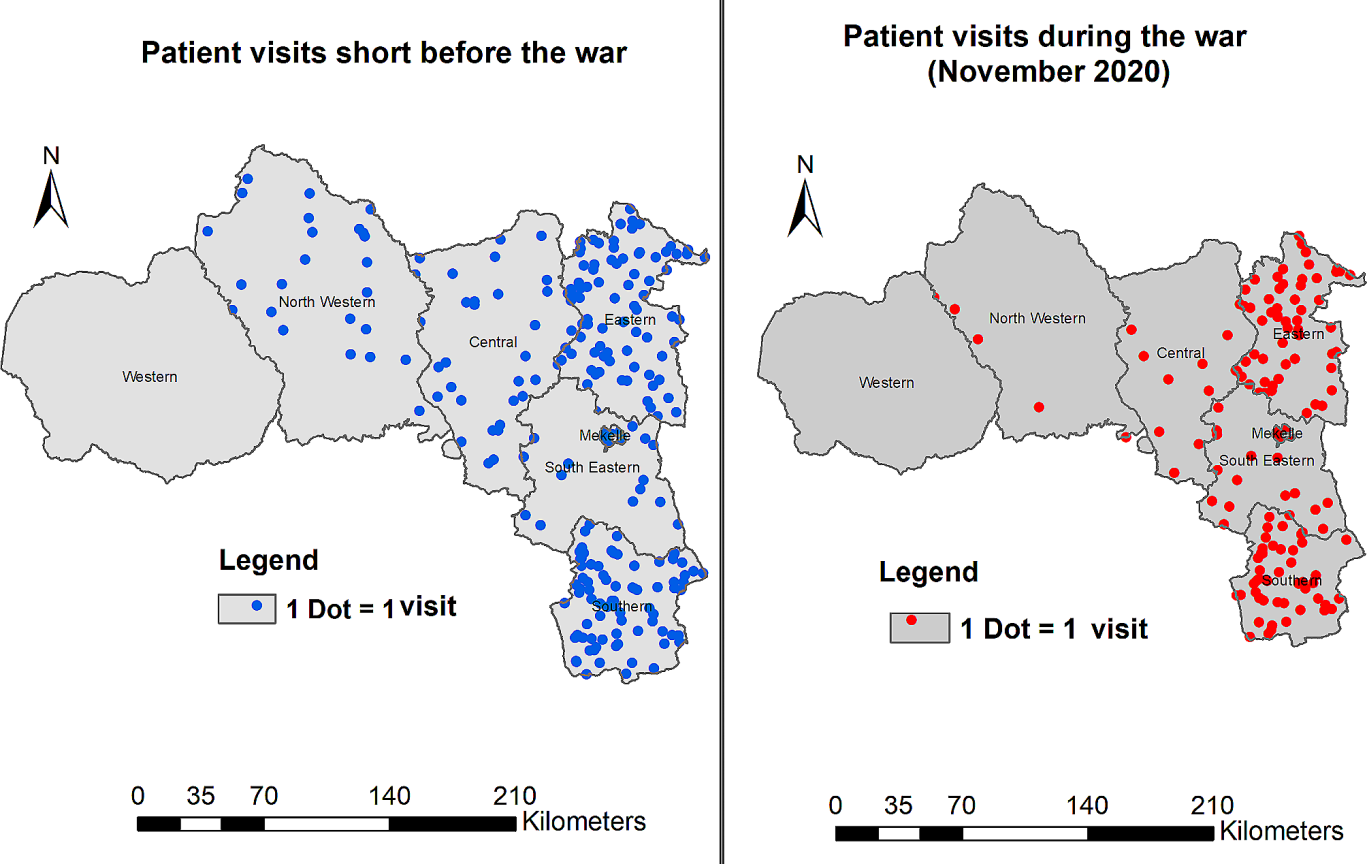
Map comparing tuberculosis patient visits shortly before (September and October average) and at the beginning of the conflict (November) in different zones of Tigray

**Fig. 4:**
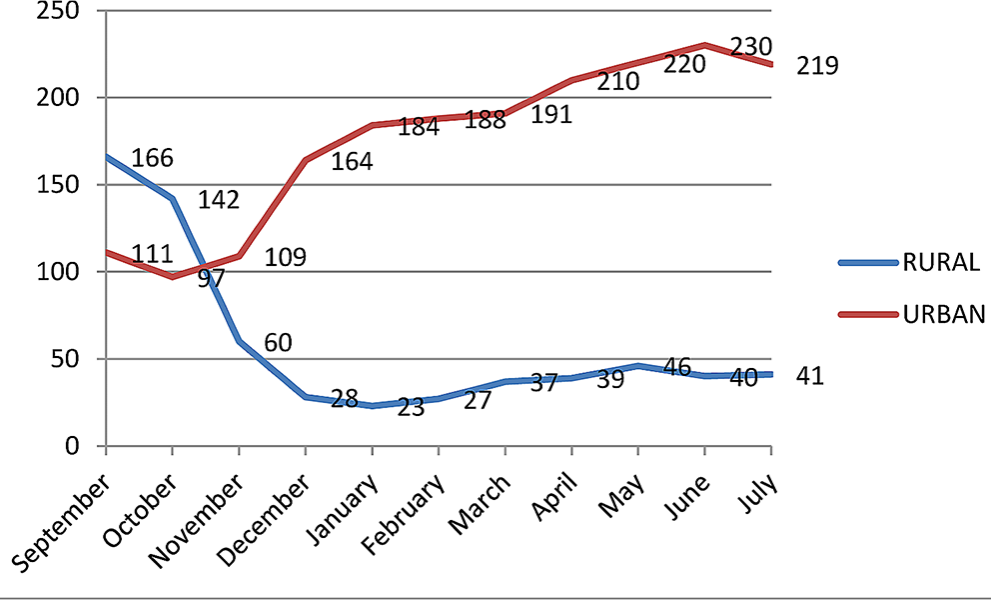
TB patient visits in rural and urban health facilities before and during the war in Tigray

Reductions were statistically significant in the central, northwestern, and eastern zones (Table 2) and were higher in the central (72%) and northwestern (79%) zones (Fig. 3). Prior to the war 60% of tuberculosis patients were served in rural clinics; this number dropped to an average of 17% during the war (Fig. 4).

**Table 2:** Comparison of TB patients who visited health facilities each month before and during the war in Tigray, stratified by zone (*n* = 50)

	Pre-war	During war			
Zone	Mean	Mean	MD	t-statistics	p – value
Central	32	10.8	-21.2	4.9	0.001
Eastern	81.5	48.6	-32.9	3.2	0.011
Mekelle	29	77.5	48.5	-2.3	0.056
Northwest	24	5.4	-18.6	5.5	< 0.001
South	77	75.1	-1.9	0.2	0.884
Southeast	14.5	6.3	-8.2	2.1	0.07

### Multidrug-resistant TB patient care

**Fig. 5:**
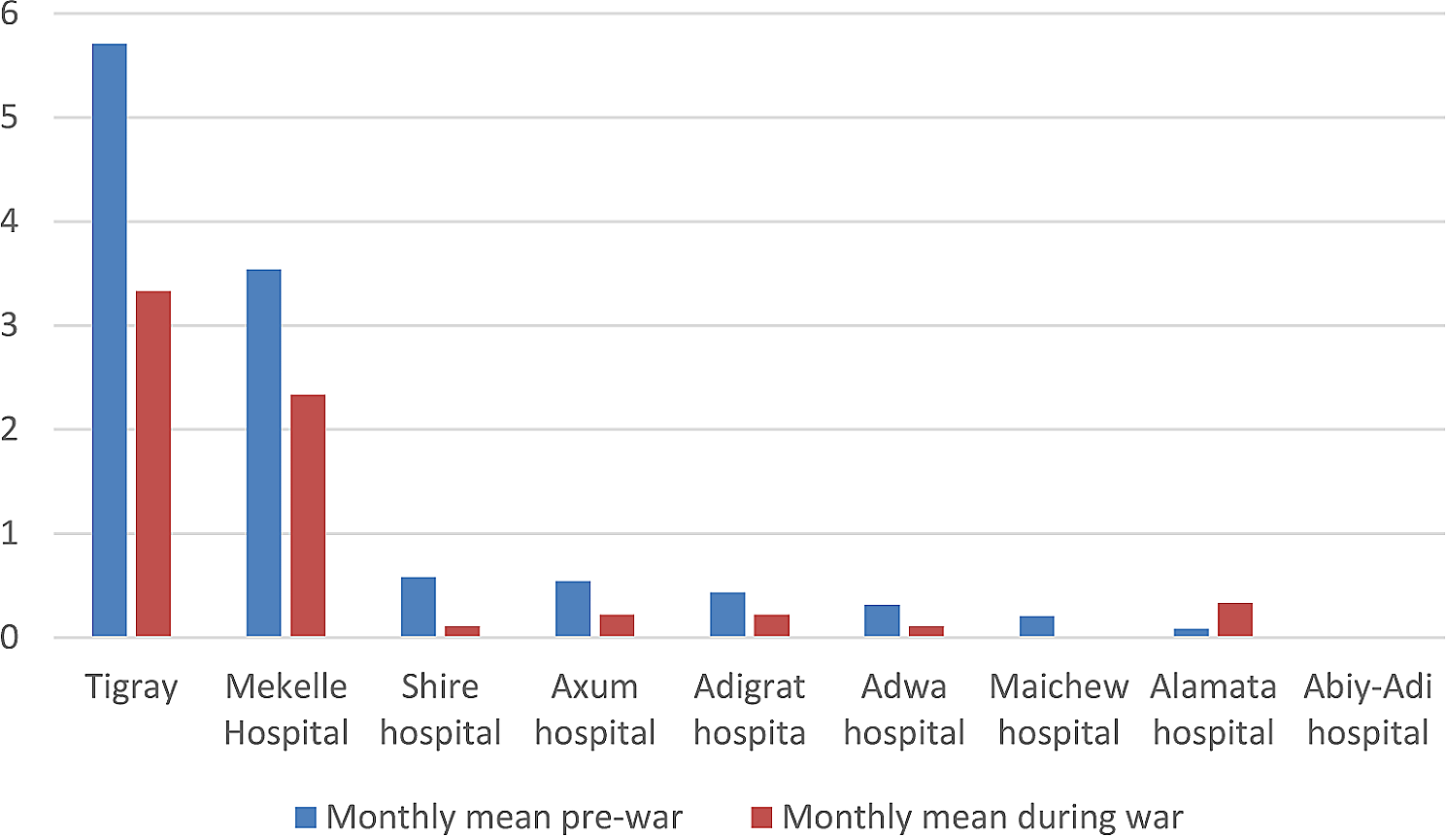
New MDR TB cases reported by health facilities before and during the war in Tigray

In comparison to the pre-war period, the monthly average number of MDR TB patients decreased by 41% during the war. This decrease was statistically significant with p-value < 0.001 (Fig. 5).

### TB diagnostic service in health facilities

The data indicated clearly that microscopes and other equipment were systematically looted. Fifty-three (69.7%), 35 (87.5%), and 15 (68%) microscopes were taken from or damaged in health centers, primary hospitals, and general hospitals, respectively, as depicted in Table 3. Acid fast bacilli (AFB) staining chemicals were unavailable in 82%, 25% and 33% of the health centers, primary hospitals, and general hospitals, respectively. In addition, 2 GeneXpert PCR machines were stolen in the study area.

**Table 3:** Comparison of laboratory equipment and reagents for TB diagnosis pre- and during the war in health facilities of Tigray (*n* = 69)

	Pre-war	During war		
	Available	Available # (%)	Looted # (%)	Damaged # (%)
Health center (*n* = 51)				
ZN staining reagent(yes)	45	8 (17.8)	37 (82.2)	0 (0.0)
Microscope(unit)	76	23 (30.3)	53 (69.7)	0 (0.0)
Slides (box)	45	21 (46.7)	24 (53.3)	0 (0.0
Primary hospital(*n* = 12)				
ZN staining reagent(yes)	12	5 (41.7)	3 (25.0)	4 (33.3)
Microscope (unit)	40	5 (12.5)	35 (87.5)	0 (0.0)
Slide (box)	600	100 (16.7)	300 (50.0)	200 (33.3)
General hospital (*n* = 6)				
ZN staining reagent(yes)	6	4 (66.7)	2 (33.3)	0 (0.0)
Microscope (unit)	22	7 (31.8)	8 (36.4)	7 (31.8)
Slide (box)	75	50 (66.7)	0 (0.0)	25 (33.3)

### Qualitative section

#### Socio-demographic status of participants

The interview participants were aged 27—60 years. Their level of education ranged from diploma to MSc/specialist physician. We interviewed a regional TB case team coordinator, a district (woreda) communicable diseases expert, an internist from a tertiary hospital, two TB focal persons from a health center and a primary hospital, a laboratory official from Tigray Health Research Institute, a laboratory technician from a primary hospital, and a medical director.

### Pre-war TB diagnosis and treatment services in Tigray

Before the war, clinical care of tuberculosis was available in all public health facilities free of charge. The private sector diagnosed 28–35% of new cases in Tigray, but once diagnosed, 80% of them were referred to public health facilities for treatment and follow-up. The Ethiopian pharmaceuticals supply agency (EPSA), a government body, supplied all TB medications and reagents every two months through its two hubs in Tigray. Prior to the war there were no major obstacles with supply.

*AFB microscopy was available in all health centers and hospitals. The 14 GeneXpert machines in the region were located in the 14 general hospitals, two referral hospitals and in THRI (the Tigray Health Research Institute).*” “*Culture was available only in THRI. Before the war, THRI received about 1000 samples annually for culture and drug susceptibility tests*.

Tuberculosis focal person in Tigray Health Research Institute.

Prior to the war Tigray enjoyed a well integrated three-tier health care system. More than 1200 health extension workers played a key role in tuberculosis case detection in the community. They identified and referred presumptive TB patients to their catchment health centers. Some 80% of Tigray’s population lives in rural areas. Health extension workers received TB patients for directly observed therapy, DOTS treatment and follow-up close to their homes.

The health extension program was first started in Tigray 20 years ago with a main focus on maternal health and infectious disease prevention and later tuberculosis was added to the package.

Tuberculosis case team coordinator from the regional health bureau.

The post office facilitated the transport of samples for GeneXpert and mycobacterial culture. Over time the case detection rate, treatment success rate and cure rate showed good improvement. All health facilities regularly reported all tuberculosis data (diagnosis, treatment and outcome) quarterly to the region’s health bureau using DHIS2.

### TB diagnosis and treatment services in Tigray during the war

When Tigray was occupied by the allied forces (mainly Ethiopian National Defense Forces and Eritrean troops), a region-wide curfew was imposed. For two months, all basic public services including telecommunication, electricity, banking and transportation were stopped. Even ambulances were not allowed to work. Many health facilities were used as military stations, ruined deliberately and looted. Many health care workers, fearing for their lives, fled.

*They used our hospital as a camp and took our microscopes.*” *“We were running for our lives, but we were visiting the hospital occasionally. Five of the 10 patients came to the hospital after some stabilization, and I told them to go to Mekelle [the city least affected by the war and 72kms far].*

A nurse from a primary hospital.

The largest hospital in Tigray, Ayder Comprehensive Specialized Hospital, located in the state capital Mekelle, was not directly attacked. But the surge of internally displaced people and lack of resources severely hampered patient care.

Our TB clinic served many patients asking for anti-TB medication but without any documents or referral. They came from the rural areas.

An internist from Ayder Hospital.

About two months after the start of the war, active war became localized to some specific areas in Tigray and many public services (telecommunication, banking and transportation) began providing limited services. After eight months of occupation, the Tigray Defense Force expelled Ethiopian National Defense Force and Eritrean troops from Tigray. The federal government responded by blocking all telecommunication, electricity, banking and transportation and imposing a complete blockade, including food and fuel. It also suspended civil servant salaries and severely restricted the supply of anti-TB and other medications.

We walked on foot to and from the health facilities because there was no transportation as a result of fuel shortage. But at least we enjoyed peace and there was no one who would kill us.

A nurse from a primary hospital.

Only moral values kept us working without any payment for 14 months.

An internist from Ayder Hospital.

### Steps towards recovery

At the time of writing, most part of Tigray is free of active conflict after 2 years of all rounded siege, though the western Tigray, some part of southern Tigray and many areas bordering Eritrea remain tenuous. Telecommunications, banking, and other public services are functioning.

Tracing patients who were lost to follow-up is a challenge. Laboratory testing is essential to check for development of drug resistance (multidrug or extensively drug resistant). For patients with MDR TB, there are no second-line medications.

We can’t talk about TB control without having even a microscope. Patients who came back after stopping medications will require either GeneXpert test or drug susceptibility tests to make sure the disease hasn’t progressed to the next stage of resistance, but we don’t have them. Currently, GeneXpert is functional only in Mekelle.

A health bureau TB coordinator.

Other interviewees were less pessimistic. An official at the THRI laboratory said they had organized mass screening in IDP centers for tuberculosis and other diseases, and they have found some TB patients lost to follow up, including MDR TB patients. These patients have been linked to facilities in Mekelle. They also hope to distribute 6000 anti-TB kits, some microscopes and reagents in collaboration with EPSA.

## Discussion

In this study, we assessed the status of clinical care related to TB services and its utilization by patients in health facilities in Tigray during the war period and compared it with the short and immediate pre-war period. The damage sustained by laboratories was assessed and challenges to the regional TB prevention and control program evaluated.

At the start of the conflict the number of TB patients using TB related medical services decreased by 34% in Tigray, by 79% in the northwest. This reduction persisted throughout the duration of the study, though it got progressively milder. A 38% increase in patient flow to Mekelle and Southeastern zones only partially compensated the 254% reduction in the other four zones. Mekelle and Southeastern zones are located in the center of Tigray and the war was most active in the peripheral part of the region. The large decrease in the detection of multidrug resistant forms of tuberculosis is very worrisome.

The primary causes of the decreased service utilization were the intentional destruction of medical facilities, the blockade of essential pharmaceuticals, the targeting of civilians and security fears, the migration of patients (both internal and abroad), and the cessation of critical public services. These results align with other studies and reports from Tigray [1, 6–9]. Health institutions in the study area have lost more than 75% of their TB laboratory diagnostic capacity with 103 of 138 microscopes having been stolen or damaged. The global objective of delivering patient-centered tuberculosis care is undermined by patients having to travel from their communities to metropolitan health institutions in order to receive medical care [10].

When TB patients cannot access appropriate care their options are limited: they can move to less affected areas, look for alternative medicine from traditional healers and religious sites, or die at home. Ethiopians frequently travel to holy water sites in search of remedies [11, 12]. These all could result in the disease spreading widely, leading to higher mortality and the emergence of drug resistance. Currently the nation has a relatively low MDR TB prevalence, just 1.1% of newly diagnosed cases and 7.5% of those who have received treatment [13–15].

Patients who migrate in large numbers to safer areas run the risk of spreading the disease during travel and when they seek shelter in internally displaced person shelters. IDP centres are too often characterized by hunger and overcrowding. Moreover, medical facilities in less impacted areas are unlikely to be able to deal effectively with a sudden surge in demand. Once in refugee camps, the stigma associated with TB is high. This can reduce patients’ health-seeking behaviour and compliance with medications [16]. These patients not only continue to transmit the disease but are also at high risk of death (up to 2/3rd of them) or severe disability [17]. In addition, a substantial proportion of MDR TB patients with primary drug resistance and associated mortality could be anticipated in the region as has been shown in previous armed conflicts [18].

Continuation of the TB program in countries with complex emergency situations is possible, as reported in different studies [16]. However, this requires a strong commitment from governmental and nongovernmental actors. During the Tigray war, no government or non-governmental agency was able to sustain the tuberculosis program in the region, despite repeated pleas from the region’s healthcare workers.

The health system in Tigray has sustained enormous damage: interruption of childhood vaccines (including vaccines against TB), migration of health care workers, destruction of health facilities, looting of medical equipment, stock-out of essential supplies, and dismantled referral and reporting system. Restoration of this system requires much work and investment. Healthcare providers who do not receive their wages for months are forced to migrate. This requires an urgent solution [19].

This study has several limitations. The clinical TB service utilization part of the study covered only the first part of the war (eight months) and excluded areas worst affected (western Tigray and villages bordering Eritrea) for security reasons. The comparative pre-war period, the two months immediately before the war, may not be representative as the looming war could have affected TB care even before the war. Any or all of these factors can lead to underestimation of the impact of the war. In addition, data from 27% of the study health facilities were unavailable due to the damage to the health facilities or TB registries and the number of TB cases in the study might have been underestimated (both before and during the war). Regarding the interviews, the process might have been influenced by the researchers’ background, experience, prior assumptions, and values. This may have shaped the conversation and affect the participants’ replies.

We propose undertaking a more comprehensive study assessing the prevalence of the disease in the population and the magnitude of lost to follow-up patients.

## Conclusion

The war has resulted in enormous disruption of TB care and requires urgent restoration. The result will be higher mortality, higher disease prevalence, and possibly different patterns of disease, including higher rates of drug resistant TB. Bringing the region back to the pre-war level of TB diagnosis, treatment, and monitoring will take many years and considerable investment.

### Electronic supplementary material

Below is the link to the electronic supplementary material.

Supplementary Material 1

Supplementary Material 2

## Data Availability

The datasets used and analyzed is put with the manuscript as an additional file.
