# Supplementary material for: War related disruption of clinical tuberculosis services in Tigray, Ethiopia during the recent regional conflict: a mixed sequential method study
Source: Confl Health. 2024 Apr 10;18:29. doi: 10.1186/s13031-024-00583-8 (PMC11005271; doi:10.1186/s13031-024-00583-8)
Supplement: Supplementary file 2 — Supplementary Material 2 [file 13031_2024_583_MOESM2_ESM.docx]

Table 1. List of codes, families, and themes

| **List of codes** | **Family** | **Theme** |
| --- | --- | --- |
| TB medication supplier; prewar supply status | Supply | Pre-war situation of TB care in Tigray |
| key player, method; target population; tests; specimen transport; private sector diagnosis rate; TB care access | TB detection |  |
| Service; location; rural population served; TB care access | Treatment |  |
| Structure; private to public referral rate | Referral |  |
| Indicator; frequency; reporting system, reported data; recipient | Monitoring and reporting |  |
| Curfew | Curfew |  |
| Telecommunication disrupted; electric city disrupted; banking disrupted; transportation disrupted  Civil servant salary suspended | Public service disruption | TB diagnosis and treatment services in Tigray during the war |
| Ambulance non-functional;  Health facilities damaged;  Healthcare workers’ fear for safety;  Ayder Hospital is operational | Healthcare system damage |  |
| TB diagnosis disrupted; TB treatment disrupted | TB service disruption |  |
| Active war localized after two months | Conflict dynamics |  |
| Active conflict is reduced in many parts of the region;  Tension remains in western Tigray; tension remains in southern Tigray; tension remains in the Eritrean border | Conflict status | Steps towards recovery |
| Public services are functional | Public service restoration |  |
| Lost to follow-up patients; drug resistance testing unavailable;  Second-line medication shortage; limited recovery progress observed | Challenges |  |
| TB screening organized in IDP centers; Identifying lost to follow-up patients; patient linkage established; anti-TB kit distribution plan; microscope and reagent distribution pan | Recovery efforts |  |
